# Bioengineered small extracellular vesicles deliver multiple SARS‐CoV‐2 antigenic fragments and drive a broad immunological response

**DOI:** 10.1002/jev2.12412

**Published:** 2024-02-09

**Authors:** Hannah K. Jackson, Heather M. Long, Juan Carlos Yam‐Puc, Roberta Palmulli, Tracey A. Haigh, Pehuén Pereyra Gerber, Jin S. Lee, Nicholas J. Matheson, Lesley Young, John Trowsdale, Mathew Lo, Graham S. Taylor, James E. Thaventhiran, James R. Edgar

**Affiliations:** ^1^ Department of Pathology University of Cambridge Cambridge UK; ^2^ Exosis, Inc. Palm Beach Palm Beach Florida USA; ^3^ Institute of Immunology and Immunotherapy University of Birmingham Birmingham UK; ^4^ MRC Toxicology Unit University of Cambridge Cambridge UK; ^5^ Cambridge Institute of Therapeutic Immunology and Infectious Disease (CITIID) University of Cambridge Cambridge UK; ^6^ Department of Medicine University of Cambridge Cambridge UK; ^7^ NHS Blood and Transplant Cambridge UK

**Keywords:** antigen, extracellular vesicles, immune presentation, SARS‐CoV‐2, vaccine

## Abstract

The COVID‐19 pandemic highlighted the clear risk that zoonotic viruses pose to global health and economies. The scientific community responded by developing several efficacious vaccines which were expedited by the global need for vaccines. The emergence of SARS‐CoV‐2 breakthrough infections highlights the need for additional vaccine modalities to provide stronger, long‐lived protective immunity. Here we report the design and preclinical testing of small extracellular vesicles (sEVs) as a multi‐subunit vaccine. Cell lines were engineered to produce sEVs containing either the SARS‐CoV‐2 Spike receptor‐binding domain, or an antigenic region from SARS‐CoV‐2 Nucleocapsid, or both in combination, and we tested their ability to evoke immune responses in vitro and in vivo. B cells incubated with bioengineered sEVs were potent activators of antigen‐specific T cell clones. Mice immunised with sEVs containing both sRBD and Nucleocapsid antigens generated sRBD‐specific IgGs, nucleocapsid‐specific IgGs, which neutralised SARS‐CoV‐2 infection. sEV‐based vaccines allow multiple antigens to be delivered simultaneously resulting in potent, broad immunity, and provide a quick, cheap, and reliable method to test vaccine candidates.

## 1 INTRODUCTION

The outbreak of the novel coronavirus SARS‐CoV‐2 and resultant global pandemic expedited the development of next‐generation vaccines. At least 40 SARS‐CoV‐2 vaccines have been approved to date, including mRNA vaccines (e.g., Pfizer‐BioNTech ‘Comirnaty’), inactivated virus vaccines (e.g., Sinopharm BIBP) and adenovirus‐based viral vector vaccines (e.g., Oxford‐AstraZeneca ‘Covishield’). The mRNA and adenovirus vaccines exclusively encode full‐length, modified versions of, or subunits of SARS‐CoV‐2 Spike protein (Stokel‐Walker, 2022)—the most highly mutated SARS‐CoV‐2 gene, and protein under highest selective pressure.

SARS‐CoV‐2 is an enveloped, positive‐stranded RNA virus with characteristic surface corona (crown) projecting from the viral surface. The corona is composed of Spike protein trimers (Li, 2016), and Spike receptor‐binding domain (sRBD) engages angiotensin‐converting enzyme 2 (ACE2) receptors on host cells to permit viral entry (Shang et al., 2020). As such, sRBD is a target for neutralising antibodies and vaccines, and anti‐sRBD antibodies potently block the entry of SARS‐CoVs to host cells (Tai et al., 2020). The Nucleocapsid (N) protein of SARS‐CoV‐2 is another major immunogen (Smits et al., 2021) and has been shown to be more stable and conserved between different strains. Whilst immunogenicity against Nucleocapsid alone will not directly neutralise the virus, anti‐N antibodies, and the generation of memory T cells, help provide long‐term immune protection. Indeed, 17 years after infection and recovery from SARS, patients possessed memory T cells reactive to N epitopes which provided protection from subsequent SARS‐CoV‐2 infection (Le Bert et al., 2020), highlighting the long‐lived memory response and cross‐reactivity against N protein. Moreover, it has been suggested that vaccination with both Nucleocapsid and Spike proteins is likely to evoke broader, stronger protection against SARS‐CoV‐2 infection (Albecka et al., 2021; Burbelo et al., 2019; Dangi et al., 2021).

Extracellular vesicles (EVs) are a diverse population of lipid‐bound particles containing a range of lipids, nucleic acids, and proteins. They are secreted by all cell types and found in all bodily fluids (Van Niel et al., 2018). The most common nomenclature divides EVs into three subclasses based on their biogenesis; (i) exosomes—vesicles of approximately 30–150 nm in diameter that originate as intraluminal vesicles (ILVs) of multivesicular bodies (MVBs) and become termed ‘exosomes’ upon fusion of the MVB with the plasma membrane; (ii) microvesicles—which form via direct outward budding of the plasma membrane and can range from 100 to 1000 nm; and (iii) apoptotic bodies—which are larger vesicles ranging between 1 and 4 μm and are formed from the fragmentation of dying cells. To clarify nomenclature, the term ‘exosome’ is used when the vesicle has been shown to originate from the endolysosomal pathway, if confirmation cannot be achieved, the term small extracellular vesicles (sEVs) is more appropriate (Théry et al., 2018).

EVs represent attractive systems for therapeutic delivery vehicles and can be used to deliver small molecules (Jang et al., 2013; Saari et al., 2015; Schindler et al., 2019), genetic material (Dooley et al., 2021; Massaro et al., 2020) and proteins (Cho et al., 2018; Fuhrmann et al., 2015). EVs may provide protection to internal cargos during transit through the extracellular space (Raposo & Stoorvogel, 2013), and EVs engage cells to promote their capture and delivery (Mulcahy et al., 2014). Therapeutic small molecules may be loaded into EVs by applying transient physical disruption of the lipid bilayer using techniques such as electroporation, freeze‐thaw, sonication or extrusion, to allow the cargo to redistribute into the particles (Liu & Su, 2019). To load proteins to EVs, genetic engineering approaches can be used to promote cargo incorporation. Commonly, proteins enriched in exosomes such as tetraspanins are employed as molecular scaffolds onto which foreign sequences can be cloned, generating chimeric proteins. This engineering approach allows for the potential targeting of EVs to specific cell types, to contain multiple foreign protein chimeras, or to deliver multiple epitopes simultaneously. This approach eliminates the manipulation steps required to load cargo after EV isolation and single EVs can be genetically engineered to contain multiple proteins or peptides to evoke multiple distinct immune responses and constitute an innovative approach for an effective virus‐free, human‐derived vaccine design.

sEVs have been used as vehicles for antigen delivery. In 1998, Zitvogel et al. demonstrated that dendritic cell‐derived sEVs carry functional Major Histocompatability Complex (MHC)‐I and MHC‐II molecules, and that these sEVs could be loaded with tumour antigens to prime T cells in vivo and suppress tumour growth (Zitvogel et al., 1998). Since then, sEV‐based immunotherapies have been investigated to treat cancers (Shi et al., 2020), and as candidate vaccines against viral (Wang et al., 2022), and parasitic diseases (Beauvillain et al., 2007; Santos & Almeida, 2021). Dendritic cell‐derived exosomes (DEXs) have perhaps received the most attention as sEV‐based therapies, and DEXs can be used to deliver peptide‐loaded MHC molecules, directly or indirectly to T cells. DEXs contain both MHC‐I and MHC‐II and are able to directly bind to and activate both CD4+ and CD8+ T cells (André et al., 2004). Alternatively, DEXs can be captured by acceptor cells, retaining and presenting peptide‐loaded MHCs (pMHCs) on the surface of cells in a process called ‘cross‐dressing’. Finally, DEXs may be internalised by APCs and passed through the endosomal pathway where pMHCs may be trafficked back to the cell surface, or peptides may be exchanged between MHCs. Alternatively, sEVs can also be simply used as vehicles to deliver antigens to APCs. Here, cell lines can be generated expressing chimeric proteins, and isolated bioengineered sEVs are able to deliver antigens to APCs and drive protective immunity (Kanuma et al., 2017; Wang et al., 2022).

In this study, we set out to determine whether genetically engineered sEVs containing multiple SARS‐CoV‐2 antigens represent a broadly applicable approach to induce immunity. To this aim, we constructed a sEV vaccine consisting of a subunit of SARS‐CoV‐2 Spike plus an antigenic region of SARS‐CoV‐2 Nucleocapsid. We show that APCs incubated with bioengineered sEVs are able to stimulate SARS‐CoV‐2 specific T cell clones. We also demonstrate that mice immunised with genetically engineered sEVs generated antigen‐specific antibodies capable of neutralising SARS‐CoV‐2 infection. Taken together, our data strengthen the potential of sEVs as vaccine platforms and validate that single sEVs can be engineered to deliver multiple distinct antigens, eliciting humoral and cellular immunological responses.

## 2 MATERIALS AND METHODS

### 2.1 Ethics statement

For human T cell work: the study was approved by the Northwest–Preston Research Ethics Committee, United Kingdom (20/NW/0240) and all participants gave written informed consent and received no compensation.

### 2.2 Cell lines and standard culture conditions

HeLa cells were a gift from Professor Scottie Robinson (CIMR, University of Cambridge, UK) and HEK293T cells were purchased from ATCC. Both cell lines were cultured in DMEM with 10% foetal bovine serum, 1% L‐glutamine, and 1% Penicillin/Streptomycin, in 5% CO_2_ at 37°C. Lymphoblastoid cell lines (LCLs) were generated by transformation of PBMCs with B95.8 strain Epstein‐Barr virus (EBV) in the presence of 1 mg/mL cyclosporin A, as previously described (Frisan et al., 2001). LCLs were maintained in RPMI supplemented with 8% FBS, 100 IU/mL penicillin and 100 g/mL streptomycin. Spike and Nucleocapsid epitope‐specific CD4+ T cell clones were isolated from SARS‐CoV‐2 convalescent healthcare workers (Tye et al., 2022) and expanded using standard methods (Long et al., 2005). All cells were regularly screened for mycoplasma tested using MycoAlert Mycoplasma Detection Kit (Lonza).

### 2.3 Generation of lentiviral expression plasmids

cDNAs were codon optimized for human cells were designed and synthesised to pUC‐Amp plasmids (Genewiz) with flanking EcoRI/BamHI restriction sites. cDNAs were cloned to the lentiviral pLVX‐Puro plasmid (Gordon et al., 2020). The viral regions selected were SARS‐CoV‐2 nucleocapsid (192–304) and sRBD (319–541 of SARS‐CoV‐2 Spike). Viral fragments were cloned to either the N terminus or to the EC1 domain (Gln36/Leu37), of CD63 and a FLAG tag was added adjacent to viral epitopes. Viral regions were flanked by Gly/Ser flexible linkers. All constructs were confirmed by Sanger sequencing and are shown in Table 1.

**TABLE 1:** Ectopic epitopes cloned into CD63 are underlined.

Construct	Amino acid sequence
CD63‐FLAG‐sRBD (C1)	MAVEGGMKCVKFLLYVLLLAFCACAVGLIAVGVGAQGGSGDYKDDDDKGGRVQPTESIVRFPNITNLCPFGEVFNATRFASVYAWNRKRISNCVADYSVLYNSASFSTFKCYGVSPTKLNDLCFTNVYADSFVIRGDEVRQIAPGQTGKIADYNYKLPDDFTGCVIAWNSNNLDSKVGGNYNYLYRLFRKSNLKPFERDISTEIYQAGSTPCNGVEGFNCYFPLQSYGFQPTNGVGYQPYRVVVLSFELLHAPATVCGPKKSTNLVKNKCVNFGGSGLVLSQTIIQGATPGSLLPVVIIAVGVFLFLVAFVGCCGACKENYCLMITFAIFLSLIMLVEVAAAIAGYVFRDKVMSEFNNNFRQQMENYPKNNHTASILDRMQADFKCCGAANYTDWEKIPSMSKNRVPDSCCINVTVGCGINFNEKAIHKEGCVEKIGGWLRKNVLVVAAAALGIAFVEVLGIVFACCLVKSIRSGYEVM*
FLAG‐N‐CD63 (C2)	MDYKDDDDKGGSGNSSRNSTPGSSRGTSPARMAGNGGDAALALLLLDRLNQLESKMSGKGQQMQQGQTVTKKSAAEASKKPRQKRTATKAYNVTQAFGRRGPEQTQGNFGDQELIRQGTDYKHWPQIGGSGMAVEGGMKCVKFLLYVLLLAFCACAVGLIAVGVGAQLVLSQTIIQGATPGSLLPVVIIAVGVFLFLVAFVGCCGACKENYCLMITFAIFLSLIMLVEVAAAIAGYVFRDKVMSEFNNNFRQQMENYPKNNHTASILDRMQADFKCCGAANYTDWEKIPSMSKNRVPDSCCINVTVGCGINFNEKAIHKEGCVEKIGGWLRKNVLVVAAAALGIAFVEVLGI VFACCLVKSI RSGYEVM*
N‐CD63‐FLAG‐sRBD (C3)	MNSSRNSTPGSSRGTSPARMAGNGGDAALALLLLDRLNQLESKMSGKGQQMQQGQTVTKKSAAEASKKPRQKRTATKAYNVTQAFGRRGPEQTQGNFGDQELIRQGTDYKHWPQIGGSGAVEGGMKCVKFLLYVLLLAFCACAVGLIAVGVGAQGGSGDYKDDDDKGGRVQPTESIVRFPNITNLCPFGEVFNATRFASVYAWNRKRISNCVADYSVLYNSASFSTFKCYGVSPTKLNDLCFTNVYADSFVIRGDEVRQIAPGQTGKIADYNYKLPDDFTGCVIAWNSNNLDSKVGGNYNYLYRLFRKSNLKPFERDISTEIYQAGSTPCNGVEGFNCYFPLQSYGFQPTNGVGYQPYRVVVLSFELLHAPATVCGPKKSTNLVKNKCVNFGGSGLVLSQTIIQGATPGSLLPVVIIAVGVFLFLVAFVGCCGACKENYCLMITFAIFLSLIMLVEVAAAIAGYVFRDKVMSEFNNNFRQQMENYPKNNHTASILDRMQADFKCCGAANYTDWEKIPSMSKNRVPDSCCINVTVGCGINFNEKAIHKEGCVEKIGGWLRKNVLVVAAAALGIAFVEVLGIVFACCLVKSIRSGYEVM*

### 2.4 Stable cell line generation

Lentiviral pLVX vectors (see above) were co‐transfected into HEK293T cells with pCMVR8.91 (‘199’) and pMD.G (VSV‐G) (‘200’) packaging plasmids using TransIT‐293 (Mirus Bio, USA). Viral supernatants were collected 48 h after transfection, passed through 0.45 μm filters to remove dead cells and debris. Recipient HeLa cells were transduced by ‘spinfection’‐ viral supernatants were centrifuged at 1800 rpm in a benchtop centrifuge at 37°C for 70 min to enhance viral transduction. Four days post‐transduction, cells were subject to antibiotic selection with 1 μg/mL puromycin for 5 days.

### 2.5 Antibodies

Antibodies used in this study are shown in Table 2.

**TABLE 2:** Antibodies used in this study.

Protein	Product number/clone name	Host species	Application	Product details
Primary antibodies				
DYKDDDDK (FLAG)	D6W5B	Rabbit	Western blot (1:1000) Immunofluorescence (1:200)	Cell signalling
SARS‐CoV‐2 Spike Receptor Binding Domain (sRBD)	S‐Ab401.1	Human	Immunofluorescence (1:100)	ALSTEM
SARS‐CoV‐2 Spike Receptor Binding Domain (sRBD)	P06DHu	Human	Immunofluorescence (1:100)	ThermoFisher
LAMP1	H4A3	Mouse	Immunofluorescence (1:200)	Developmental Studies Hybridoma Bank
CD63	H5C6	Mouse	Western blot (1:1000), Immunofluorescence (1:200)	BioLegend
CD9	Ab92726	Rabbit	Western blot (1:1000)	Abcam
GAPDH	2118S	Rabbit	Western blot (1:2000)	Cell signalling
Syntenin	Ab133267	Rabbit	Western blot (1:1000)	Abcam
Tsg101	4A10	Mouse	Western blot (1:1000)	GeneTex
ALIX	3A9	Mouse	Western blot (1:1000)	GeneTex
Calnexin	Ab133615	Rabbit	Western blot (1:1000)	Abcam
FLAG (APC)	637307	Rat IgG2aλ	Flow cytometry (1:1000)	BioLegend
CD63 (FITC)	353005	Mouse IgG1κ	Flow cytometry (1:1000)	BioLegend
IgG2aλ, isotype control, Rat APC	402305	Rat IgG2aλ	Flow cytometry (1:1000)	BioLegend
IgG1κ, isotype control, Mouse, FITC	400107	Mouse IgG1κ	Flow cytometry (1:1000)	BioLegend
Secondary antibodies				
Anti‐rabbit IgG Alexa Fluor 555	A‐21428	Goat	Immunofluorescence (1:200)	ThermoFisher
Anti‐mouse IgG Alexa Fluor 488	A‐11001	Goat	Immunofluorescence (1:200)	ThermoFisher
Anti‐mouse IgG Alexa Fluor 555	A‐21422	Goat	Immunofluorescence (1:200)	ThermoFisher
Anti‐mouse IgG Alexa Fluor 647	A‐21235	Goat	Immunofluorescence (1:200)	ThermoFisher
Anti‐human IgG Alexa 488	A‐11013	Goat	Immunofluorescence (1:200)	ThermoFisher
Anti‐rabbit IRDye 680	926‐68071	Goat	Western blot (1:10,000)	Li‐Cor
Anti‐mouse IRDye 800	926‐32210	Goat	Western blot (1:10,000)	Li‐Cor
Anti‐human IRDye 680	926‐68078	Goat	Western blot (1:10,000)	Li‐Cor

### 2.6 Western blotting

Washed cells or sEV‐enriched pellets were lysed in lysis buffer (1% Triton‐X100, 1 mM EDTA, 150 mM NaCl, 20 mM Tris pH 7.5) supplemented with 1× cOmplete™ EDTA‐free Protease Inhibitor Cocktail (11836170001, Roche) on ice for 30 min with regular vortexing. Lysates were centrifuged at 14,000×*g* for 15 min at 4°C. Resultant lysates were mixed with 4× NuPage LDS sample buffer (ThermoFisher), boiled at 65°C or 95°C for 10 min and subjected to SDS‐PAGE on NuPage 4%–12% Bis‐Tris precast gels (ThermoFisher). Samples were transferred to PVDF membranes, blocked with 5% milk/PBS‐Tween and probed with the above primary and secondary antibodies. To visualise proteins, membranes were imaged using an Odyssey CLx (Li‐Cor).

### 2.7 Flow cytometric analysis

Cells were gently trypsinised and centrifuged at 300×*g* in a benchtop centrifuge at 4°C for 5 min to wash cells. Subsequently, cells were blocked in 1% BSA/PBS for 30 min on ice. Surface staining with fluorophore‐conjugated antibodies was performed in PBS with 0.5% BSA + 1 mM EDTA for 30 min on ice. For intracellular staining, cells were fixed and permeabilised using 0.1% saponin/PBS, followed by blocking in 1% BSA/PBS for 30 min on ice. Staining with fluorophore‐conjugated antibodies was performed in PBS with 0.5% BSA + 1 mM EDTA for 30 min on ice. Cells were then washed in cold PBS twice and the fluorescence intensity was measured on a four laser Cytoflex S (Beckman coulter, 488, 640, 561, 405 nm).

### 2.8 Immunofluorescence

Cells were seeded to glass coverslips and left overnight to adhere. The following day, cells were fixed with 4% PFA/PBS. Cells were quenched with 15 mM glycine/PBS and permeabilised using 0.1% saponin/PBS. Blocking and subsequent antibody labelling were performed with 1% BSA supplemented with 0.01% saponin/PBS. Cells were mounted on slides with mounting medium containing DAPI (Invitrogen) and images were acquired using a LSM700 confocal microscope (40×, 1.3 nA or 63×, 1.4 nA, oil immersion objectives; ZEISS).

### 2.9 sEV isolation

sEV‐enriched preparations were generated from cell culture supernatants as previously described (Théry et al., 2006). Briefly, cells were grown in equal numbers on 245 mm × 245 mm dishes in 60 mL of sEV‐depleted media. Cells were treated with 100 nM Bafilomycin A1 for 16 h to induce sEV release. Supernatants were collected and large cell debris removed by centrifugation at 300×*g*, 10 min. Pellets were discarded, and supernatants were then spun at 2000×*g*, 10 min to remove smaller cell debris. Pellets were discarded and supernatants were ultracentrifuged (Type 70 Ti Rotor, Beckman Coulter) at 10,000×*g*, 30 min to remove larger extracellular vesicles. Pellets were discarded and supernatants were again collected and ultracentrifuged at 100,000×*g*, 70 min. Pellets were washed in PBS and re‐pelleted at 100,000×*g*, 70 min.

### 2.10 Transmission electron microscopy of isolated sEVs

To visualise sEVs, vesicle pellets were resuspended on to formvar/carbon coated Cu‐Pd 200 mesh grids (Agar Scientific) and incubated for 20 min, followed by fixation with 2% PFA/PBS for 20 min. Grids were washed five times with H_2_O and negative contrast was achieved by incubating the grid for 10 min in 0.4% uranyl acetate/methylcellulose.

To immunolabel sEVs, vesicle pellets were resuspended on to formvar/carbon coated Cu‐Pd 200 mesh grids (Agar Scientific) for 20 min and fixed as above. Grids were blocked with 1% BSA/PBS and incubated with primary antibodies (Section 2.6) for 45 min, washed five times with 0.1% BSA/PBS and incubated with Protein‐A Gold 10 nm (Utrecht University, the Netherlands) for 20 min. Grids were washed with PBS and fixed with 1% glutaraldehyde/PBS for 5 min then washed with H_2_O. Negative staining was performed as above. To visualise samples, an FEI Tecnai transmission electron microscope was used at an operating voltage of 80 kV.

### 2.11 Surface labelling immunogold transmission electron microscopy

Cells were grown on Thermanox coverslips (Nunc) and left overnight to adhere. Cells were fixed with 8% PFA/0.1 M cacodylate buffer (pH 7.4) at a 1:1 ratio with culture medium (final concentration 4% PFA) for 20 min before being quenched with 20 mM glycine/0.1 M cacodylate buffer. Cells were blocked with 1% BSA/PBS before being incubated with primary antibodies (Section 2.6) for 1 h at room temperature. The cells were then labelled with 10 nm Protein‐A Gold (Utrecht University, the Netherlands), and re‐fixed with 2% PFA, 2.5% glutaraldehyde/0.1 M cacodylate, before being post‐fixed with 1% osmium tetroxide: 1.5% potassium ferrocyanide. Samples were contrast enhanced using 1% tannic acid/0.1 M cacodylate buffer. Samples were washed with water before being dehydrated with an ethanol series. Epoxy propane was used to infiltrate cells (CY212 Epoxy resin (Agar Scientific): propylene oxide (Agar Scientific)) before being infiltrated with neat CY212 Epoxy resin. Coverslips were mounted to pre‐baked resin stubs and polymerised overnight at 65°C. Thermanox coverslips were removed with a heat block. 70 nm sections were cut using a Diatome diamond knife mounted to an ultramicrotome and were collected to formvar coated grids before being stained with lead citrate. An FEI Tecnai transmission electron microscope was used to visualise samples at an operating voltage of 80 kV.

### 2.12 NanoFCM

sEV samples were diluted 1:10–1:100 and analysed using the Flow Nano Analyzer (NanoFCM), according to the manufacturers protocol (Tian et al., 2020). Briefly, lasers were calibrated using 200 nm control beads (NanoFCM Inc.), which were analysed as a reference for particle concentration. A mixture of various sized beads (NanoFCM Inc.) were analysed to set a reference for size distribution. PBS was analysed as background signal. Particle concentration and size distribution were calculated using NanoFCM software (NanoFCM profession V1.0) and normalised to cell number and dilution necessary for adequate NanoFCM‐reading.

### 2.13 FLAG and sRBD enzyme‐linked immunosorbent assays (ELISA)

FLAG and sRBD concentrations in both lysed and non‐lysed cells and sEVs were determined, by using DYKDDDDK‐Tag Protein ELISA Kit (Abcam, ab285234) and Human SARS‐CoV‐2 RBD ELISA Kit (ThermoFisher), respectively, and were used according to the manufacturer's instructions.

For non‐lysed cells, cells were trypsinised and collected from 9 cm dishes and pelleted by centrifugation (300×*g*, 5 min). Cell pellets were resuspended in 1 mL PBS and samples applied to ELISA plates. For lysed cells, cells were grown in 9 cm dishes and scraped in 1 mL lysis buffer plus protease inhibitors (as for western blotting, Section 2.6). Cell lysates were applied to ELISA plates.

For non‐lysed sEVs, sEV‐enriched pellets (see Section 2.9) were resuspended in 1 mL PBS. For lysed sEVs, sEV‐enriched pellets (see Section 2.9) were resuspended in 1 mL Lysis buffer plus protease inhibitors (as for western blotting, Section 2.6).

Samples were quantified using corrected values of 450 and 650 nm, reading absorbance in a microplate reader (BMG LUMIstar Omega). A 4‐parameter logistic curve was used to describe the data. For each experiment at least two replicates were used for analysis.

### 2.14 Cell clone assay

CD4^+^ T cell clones were isolated from polyclonal cultures as previously described (Tye et al., 2022). CD4+ T cell clones (2000 cells per well) were incubated in V‐bottom 96‐well microtest plate wells with 5 × 10^4^ cells per well of autologous LCL or allogeneic LCL expressing the relevant HLA class II restriction allele. LCLs were exposed to either; 5 μg/mL purified epitope peptide (>85% purity, Alta Biosciences, UK), 5–100 μg/mL recombinant sRBD or N protein (Abcam, ab288548 and ab279288), 5–100 μg/mL of purified sEVs preparations, or equivalent volumes of PBS (negative control) for 3 h, washed and added to T cells. After 18 h of co‐culture, the culture supernatant medium was harvested and the IFN‐γ release into the supernatant was tested by ELISA according to the manufacturers protocol (Invitrogen). Values were calculated as a percentage of IFN‐γ released in response to the LCL plus peptide control.

### 2.15 Mouse immunisation and serum collection

For in vivo experiments, sex‐mixed C57BL/6 mice aged 8–12 weeks (*n* =  5 per group) were inoculated subcutaneously with 5 μg Spike protein adjuvanted with AddaVax (oil‐in‐water squalene solution), 100 μL C3 sEVs or 100 μL WT sEVs on days 0, 14, and 28. Blood samples (100 μL) were collected from mice on days 14 and 28 and serum was isolated. On day 48, mice were killed and serum and spleens were collected immediately.

### 2.16 Live virus neutralisation assays

The SARS‐CoV‐2 used in this study was a wildtype (lineage B) virus (SARS‐CoV‐2/human/Liverpool/REMRQ0001/2020, a kind gift from Ian Goodfellow, University of Cambridge), isolated by Lance Turtle (University of Liverpool) and David Matthews and Andrew Davidson (University of Bristol) (Moore et al., 2020; Patterson et al., 2020; Daly et al., 2020). Luminescent HEK293T‐ACE2‐30F‐PLP2 reporter cells (clone B7) expressing ACE2 and SARS‐CoV‐2 Papain‐like protease‐activatable circularly permuted firefly luciferase (FFluc) were from the National Institute for Biological Standards and Control (NIBSC, 101062) (Gerber et al., 2022).

Sera were heat‐inactivated at 56°C for 30 min before use, and neutralising antibody titres at 50% inhibition (NT50s) measured as previously described (Bergamaschi et al., 2021, Gerber et al., 2022, van der Klaauw et al., 2023). In brief, luminescent reporter cells were seeded in flat‐bottomed 96‐well plates overnight. SARS‐CoV‐2 viral stock (MOI = 0.01) was pre‐incubated with a 3‐fold dilution series of each serum for 2 h at 37°C, then added to the cells. 16 h post‐infection, cells were lysed in Bright‐Glo Luciferase Buffer (Promega) diluted 1:1 with PBS and 1% NP‐40, and FFluc activity measured by luminometry. Experiments were conducted in duplicate.

To obtain NT50s, titration curves were plotted as FFluc versus log (serum dilution), then analysed by non‐linear regression using the Sigmoidal, 4PL, X is log(concentration) function in GraphPad Prism. NT50s were reported when (1) at least 50% inhibition was observed at the lowest serum dilution tested (1:10), and (2) a sigmoidal curve with a good fit was generated. For purposes of visualisation and ranking, samples for which visual inspection of the titration curve indicated inhibition at low dilutions, but which did not meet criteria (1) and (2) above, were assigned an arbitrary NT50 of 4.

### 2.17 Quantification of sRBD and nucleocapsid IgG titre in vivo

Quantitative IgG antibody titres against SARS‐CoV‐2 sRBD and N proteins were measured using IgG titre serological (mouse anti‐SARS‐CoV‐2 Spike RBD antibody IgG titre serological assay kit or anti‐SARS‐CoV‐2 Nucleocapsid antibody IgG titre serological assay kit, Acro), according to the manufacturer's instructions. In brief, 96‐well plates were blocked and washed. Samples were prediluted 1:1000 in sample diluent and added to the wells in duplicate alongside the reference standard and assay kit controls. Following incubation, washing and addition of anti‐IgG detection antibodies, stop solution buffer was added to all wells and plates were measured immediately using a plate reader (BMG LUMIstar Omega). Absorbance (OD) was calculated as the absorbance at 450 nm minus the absorbance at 630 nm to remove background prior to statistical analysis. The OD value reflects the amount of antibody bound and was used to calculate IgG concentrations.

### 2.18 Statistical analysis

Results are shown as mean ± SEM of the indicated number of independent experiments. The statistical significance of differences in group results was compared using one‐way analysis of variance (ANOVA) with multiple comparison testing as indicated. All statistical analyses and plots were carried out using GraphPad Prism 9 (GraphPad Software Inc., La Jolla, CA, USA) unless stated otherwise. The number of biological samples, corresponding statistical test and significance levels are indicated in each figure legend.

## 3 RESULTS

### 3.1 Design for generation of SARS‐CoV‐2 antigen‐containing EVs

All current COVID‐19 mRNA and adenovirus‐based viral vector vaccines encode SARS‐CoV‐2 Spike, or derivatives thereof. Here, we sought to examine whether sEVs could be bioengineered to harbour multiple viral subunits or antigenic regions from SARS‐CoV‐2, individually or in combination, and to determine their ability to generate immune responses.

**FIGURE 1:**
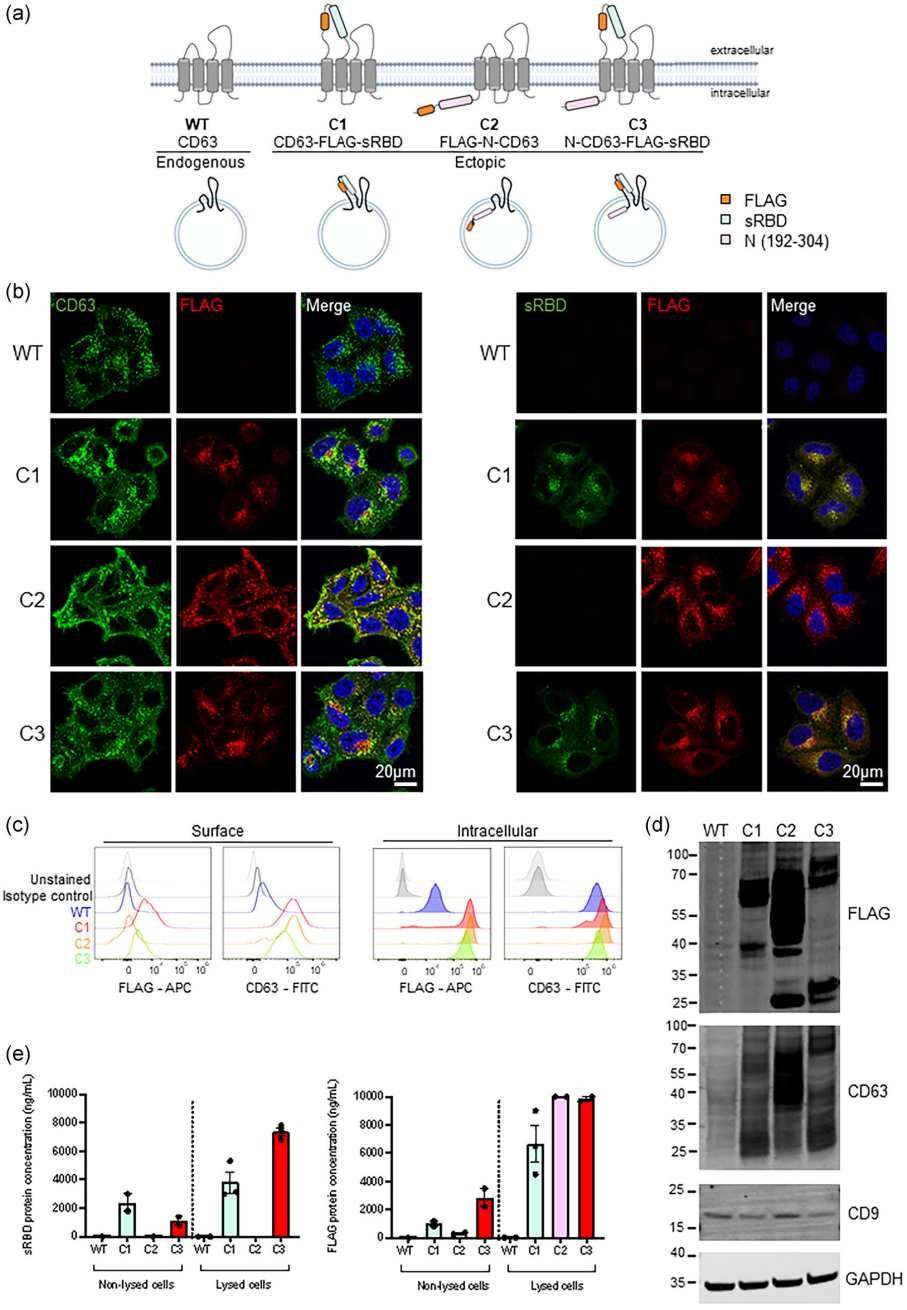
Design of CD63‐based constructs and cell line verification. (a) A schematic representation of CD63 fusion proteins. Three CD63 fusion constructs were designed; one containing sRBD (residues 319–541 of SARS‐CoV‐2 Spike) within EC1 of CD63 (outside of sEVs), one comprising an antigenic fragment of nucleocapsid (N) protein (residues 192–304) at the N‐terminus of CD63 (inside of sEVs), and one containing both sRBD (outside) and N regions (inside). These were designated C1, C2, C3, respectively. A FLAG epitope was incorporated alongside the fusion constructs. (b) Confocal immunofluorescence of WT (mock), C1, C2 or C3 stable cells stained with anti‐CD63 (green), anti‐FLAG (red) and DAPI (blue), or anti‐sRBD (S‐Ab401.1) (green), anti‐FLAG (red) and DAPI (blue). Scale bars = 20 μm. Representative images from at least three independent experiments. (c) Surface (non‐permeabilised) and intracellular (permeabilised) flow cytometry analysis was performed on WT (mock), C1, C2 or C3 stable cell lines using antibodies against CD63 and FLAG. (d) Western blot analysis of lysates from WT (mock), C1, C2 and C3 cells. Blots were probed with anti‐FLAG, anti‐CD63, anti‐CD9 and anti‐GAPDH (loading control) antibodies. Representative images from three independent experiments. (e) FLAG and sRBD enzyme‐linked immunosorbent assays (ELISA) were performed on non‐lysed or lysed WT (mock), C1, C2 and C3 cells. *n* = ≥2; mean ± SEM.

To produce bioengineered EVs, fusion constructs were designed encoding the EV‐enriched scaffold CD63, and antigenic regions of SARS‐CoV‐2. CD63 belongs to the tetraspanin protein family and is composed of short N‐ and C‐ termini, four transmembrane domains, and two extracellular domains, the first being short (EC1) and the second larger (EC2) (Boucheix & Rubinstein, 2001). At steady‐state, CD63 is enriched in the endocytic pathway, and specifically on intraluminal vesicles (ILVs) of multivesicular bodies (MVBs) (Andreu & Yáñez‐Mó, 2014). Fusion of MVBs with the plasma membrane liberates these ILVs where they become termed exosomes. CD63 has been used as a marker of exosomes for the last two decades, due to its enrichment in small EVs as compared to whole cell lysates, and to the steady‐state accumulation of CD63 in MVBs (Escola et al., 1998, [Link]). As a result, CD63 constructs have been widely used to label and track exosomes in vitro and in vivo (Gupta et al., 2020). Fluorescent proteins have been tagged to both the cytosolic N terminus (Harrison‐Lavoie et al., 2006), and to the first extracellular domain (EC1) of CD63 (Sung et al., 2020) without deleteriously impacting the stability or trafficking of CD63. Accordingly, fusion constructs consisting of SARS‐CoV‐2 epitopes were cloned to the N terminus or first extracellular loop (EC1) of CD63 (Gln36/Leu37), allowing viral epitopes to be placed on the inside or outside of sEVs, respectively (Table 1). Epitope position on the inside or outside of sEVs was selected based on the natural topology—ensuring native glycosylation and posttranslational modifications. Three CD63‐based constructs were designed; one containing sRBD (residues 319–541 of SARS‐CoV‐2 Spike) within EC1 of CD63 (outside of sEV), one comprising a region of nucleocapsid (N) protein (residues 192–304) at the N‐terminus of CD63 (inside of sEV), and one containing both sRBD (outside) and N regions (inside). These were designated C1, C2, C3, respectively (Figure 1a). An extended polypeptide of Nucleocapsid, rather than full length Nucleocapsid was used as full length Nucleocapsid‐CD63 constructs were found to express poorly and mislocalise (data not shown). Viral polypeptides were flanked by glycine/serine flexible linkers and a FLAG epitope tag was incorporated alongside the fusion construct to aid determination of ectopic expression.

### 3.2 Establishing and verification of EV producing cells

Stable cell lines expressing FLAG‐tagged CD63‐fusion constructs were generated by lentiviral transduction. HeLa cells were selected to express fusion constructs due to their genomic plasticity, ease of handling and ability to thrive in chemically defined media formulations which streamline EV production. We, and others, have shown that Bafilomycin A can be used as a tool to enhance CD63‐positive sEV release from HeLa cells (Edgar et al., 2016; Mathieu et al., 2021). Additionally, recent work has demonstrated the use of HeLa cells as a source of genetically engineered EVs (Costa Verdera et al., 2017; O'Brien et al., 2022). Immunofluorescence confirmed ectopic FLAG expression in all three cell lines which colocalised with CD63 in punctate organelles (Figure 1b, Figure S1A). CD63 expression was enhanced in all three cell lines due to the additional ectopic expression of CD63 but importantly this overexpression of CD63 did not impact its distribution. sRBD labelling was observed in both C1 and C3 cells where it colocalised with FLAG staining but was not detected in mock or C2 cell lines as expected. FLAG staining colocalised with both CD63 and LAMP1 for all three cell lines (Figure S1B), demonstrating the steady‐state localisation of fusion constructs to the late endosomal pathway.

The correct topology of CD63 fusion constructs was confirmed in cells by surface and intracellular flow cytometry (Figure 1c). The surface levels of CD63 were higher in C1, C2 and C3 than in mock cells, reflecting the additional ectopic expression of CD63 in all three cell lines. By surface staining, only cells ectopically expressing FLAG tags on the luminal (extracellular) domain of CD63 (C1 and C3) displayed anti‐FLAG staining. Anti‐FLAG staining could only be observed in C2 cells upon permeabilization, and C1, C2 and C3 cell lines were found to express FLAG to similar levels. Western blotting of C1, C2 and C3 cell lysates confirmed the expression of ectopic protein, and resultant increase in the molecular weight of CD63 (Figure 1d). CD63 expression was significantly higher in C1, C2 and C3 lysates relative to WT cells (Figure S1C). The presence of FLAG from all three stable cell lines was also confirmed. C2 lysates reproducibly produced very strong bands by western blotting, and this is likely due to the different affinity that anti‐FLAG antibodies have to terminal and internal FLAG epitopes. FLAG and CD63‐positive bands were observed at ∼25 kDa in bioengineered cell lines (Figure 1d). We speculated that these bands may be caused by lysosomal degradation of our fusion proteins. Inhibition of lysosomal proteolysis using leupeptin caused these bands to diminish, highlighting lysosomal degradation as a source of these bands (Figure S1D). No significant difference in CD9, a related tetraspanin was observed.

Finally, to verify the topology and expression in C1, C2 and C3 stable cells, enzyme‐linked immunosorbent assays (ELISA) for FLAG and sRBD were performed on equal number of whole (non‐lysed) cells or lysed cells (Figure 1e). Although the steady‐state distribution of CD63 is largely endosomal, some is localized to the plasma membrane (Mathieu et al., 2021), and levels are likely increased by overexpression. FLAG and sRBD were detected by ELISA from C1 and C3 cells, but neither were detected from WT or C2 cells. Lysis of samples allowed FLAG to be detected in C2 cells, and lysis greatly increased the ability of anti‐FLAG antibodies to bind. Reassuringly, both ELISAs gave similar concentrations of sRBD and FLAG, providing us with confidence that these ELISAs would also be suitable for analysing protein concentrations from sEVs.

### 3.3 Characterisation of genetically engineered EVs

**FIGURE 2:**
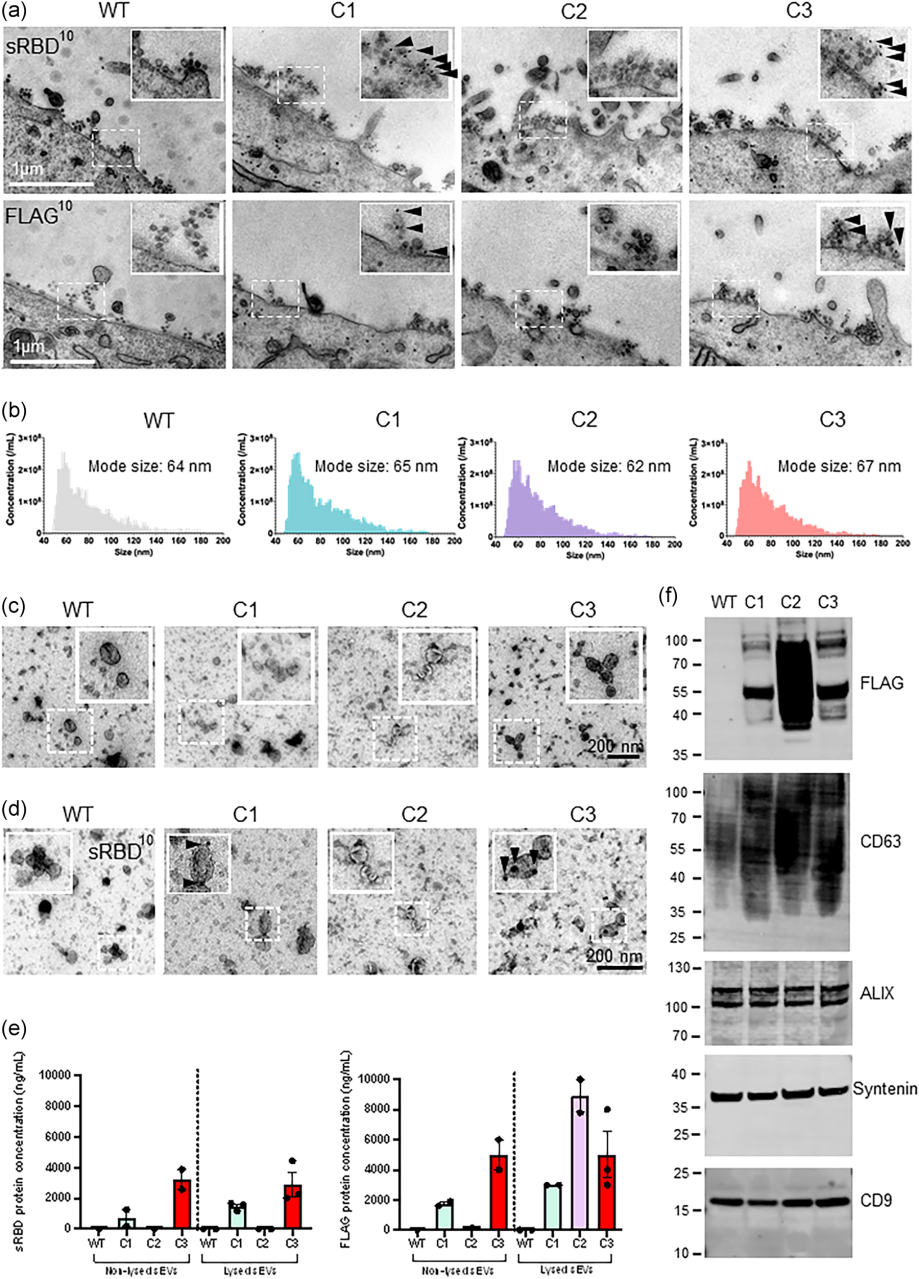
Characterisation of bioengineered sEVs. (a) Surface immunogold electron microscopy was performed on WT (mock), C1, C2 or C3 stable cells. Cells were treated with BafA1 (100 nM) for 16 h to induce sEV release. Samples were labelled with anti‐FLAG or anti‐sRBD antibodies, followed by 10 nm Protein‐A gold. Scale bar = 1 μm. Magnification (×1.5) shows the presence of labelled sEVs near the plasma membrane. (b) Size and concentration of sEVs isolated from WT, C1, C2 and C3 cell lines and analysed by NanoFCM. *n* = 3 independent experiments. (c) Transmission electron microscopy of isolated sEVs from WT (mock), C1, C2 or C3 cells. Scale bars = 200 nm. Insets are magnified 2×. (d) Immuno electron microscopy micrograph of isolated sEVs from WT, C1, C2, C3 cells labelled with an anti‐sRBD antibody. Arrowheads indicate gold labelling. Scale bar = 200 nm. (e) FLAG and sRBD enzyme‐linked immunosorbent assays (ELISA) was performed on non‐lysed and lysed stable cells. *n* = ≥2; mean ± SEM. (f) sEVs isolated from WT, C1, C2 and C3 stable cells were analysed by Western blot. FLAG staining was used to demonstrate the presence of engineered protein in sEVs. The sEV positive markers CD63, ALIX, Syntenin and CD9 were blotted. Representative images from three independent experiments.

HeLa cells express abundant levels of tetherin, and as a result a proportion of exosomes remain attached to the plasma membrane following MVB‐plasma membrane fusion (Edgar et al., 2016). We took advantage of the presence of tethered exosomes and applied surface labelling transmission electron microscopy (TEM) to cells—using the plasma membrane as an internal control for immunolabelling. Mock, C1, C2 or C3 cell lines were treated with Bafilomycin A for 16 h to induce sEV release (Edgar et al., 2016), and cells were subsequently fixed and labelled with anti‐FLAG or anti‐sRBD antibodies. In this method, fixed cells are labelled with antibodies raised against epitopes on the luminal domain of proteins, and the relative enrichment of labelling on sEVs can be compared with plasma membrane staining (see methods 3.11). In both C1 and C3 cells, anti‐FLAG and anti‐sRBD antibodies decorated sEV clusters associated with the plasma membrane but did not stain the plasma membrane itself (Figure 2a, Figure S2A, B). Mock and C2 sEVs (where FLAG epitope was expressed on the inside of the sEVs) were devoid of labelling, confirming the absence of external sRBD/FLAG. To further characterize sEVs, supernatants were collected from Bafilomycin A treated cells and sEVs were analysed by flow nanoanalyzer (NanoFCM)—a cytometer designed to analyse particles smaller than the wavelength of visible light. NanoFCM revealed there was no difference in the average diameter or size distribution of engineered sEVs compared to WT sEVs (Figure 2b). In addition, isolated sEVs were characterized by negative stain TEM. Micrographs of isolated sEVs revealed a heterogeneous population of ‘cup‐shaped’ spherical vesicles characteristic of sEVs (Figure 2c). Immunogold TEM was performed on isolated sEVs using anti‐sRBD antibodies, again confirming the external exposure of sRBD on C1 and C3 sEVs (Figure 2d).

To further confirm the topology of bioengineered sEVs and to determine the concentration of ectopic proteins, ELISAs were performed on independent sEV‐enriched preparations. ELISAs performed in non‐lysed conditions, allowed for the discrimination of external and internal inserts. ELISAs performed in non‐lysed conditions calculated sRBD mean concentrations for C1 sEVs to be 715.3 ng/mL (SEM; 539.3 ng/mL), and for C3 sEVs to be 3222.4 ng/mL (SEM; 654.5 ng/mL). Lysis of sEVs did not greatly affect sRBD ELISA values (C1, 1481.2 ng/mL (SEM 147.7 ng/mL); C3, 2897.3 ng/mL (SEM 764.8 ng/mL)). Reassuringly, neither WT (mock) sEVs nor C2 sEVs recorded detectable levels of sRBD (Figure 2e).

Similarly, ELISAs were performed to determine the concentrations of FLAG with sEV samples. In non‐lysed sEVs, C1 sEVs contained 1750 ng/mL (SEM; 212.1 ng/mL) and C3 sEVs 5000 ng/mL (SEM; 1414 ng/mL). Lysis of sEVs allowed the concentration of all three sEVs to be analysed (C2 FLAG epitope is found on the inside of sEVs), and C2 sEVs were found to contain 8900 ng/mL (SEM; 155 ng/mL) of FLAG. WT (mock) sEVs did not record detectable levels of FLAG. It should be noted that anti‐FLAG antibodies have differing affinities for FLAG epitopes depending on whether the epitopes are positioned at N‐ or C‐termini or are found internally within a protein (as also observed by western blotting), explaining potential higher quantified levels of C2 versus C1 or C3 sEVs.

Western blotting of isolated sEV preparations from mock, C1, C2, and C3 cells verified the presence of FLAG in sEVs from all three engineered, stable cell lines (Figure 2f). Likewise, CD63 was highly enriched from C1, C2 and C3 sEVs compared to WT sEVs, again reflecting the additional ectopic expression of CD63 (Figure 2f, Figure S2C). sEV preparations were determined to be enriched for the EV‐enriched proteins ALIX, syntenin, CD9 and Tsg101, but not for the ER marker Calnexin (Théry et al., 2018).

Collectively, these data confirm the production of engineered sEVs containing multiple viral antigenic regions with correct topology.

### 3.4 APCs primed with bioengineered sEVs can activate CD4+ T cells

**FIGURE 3:**
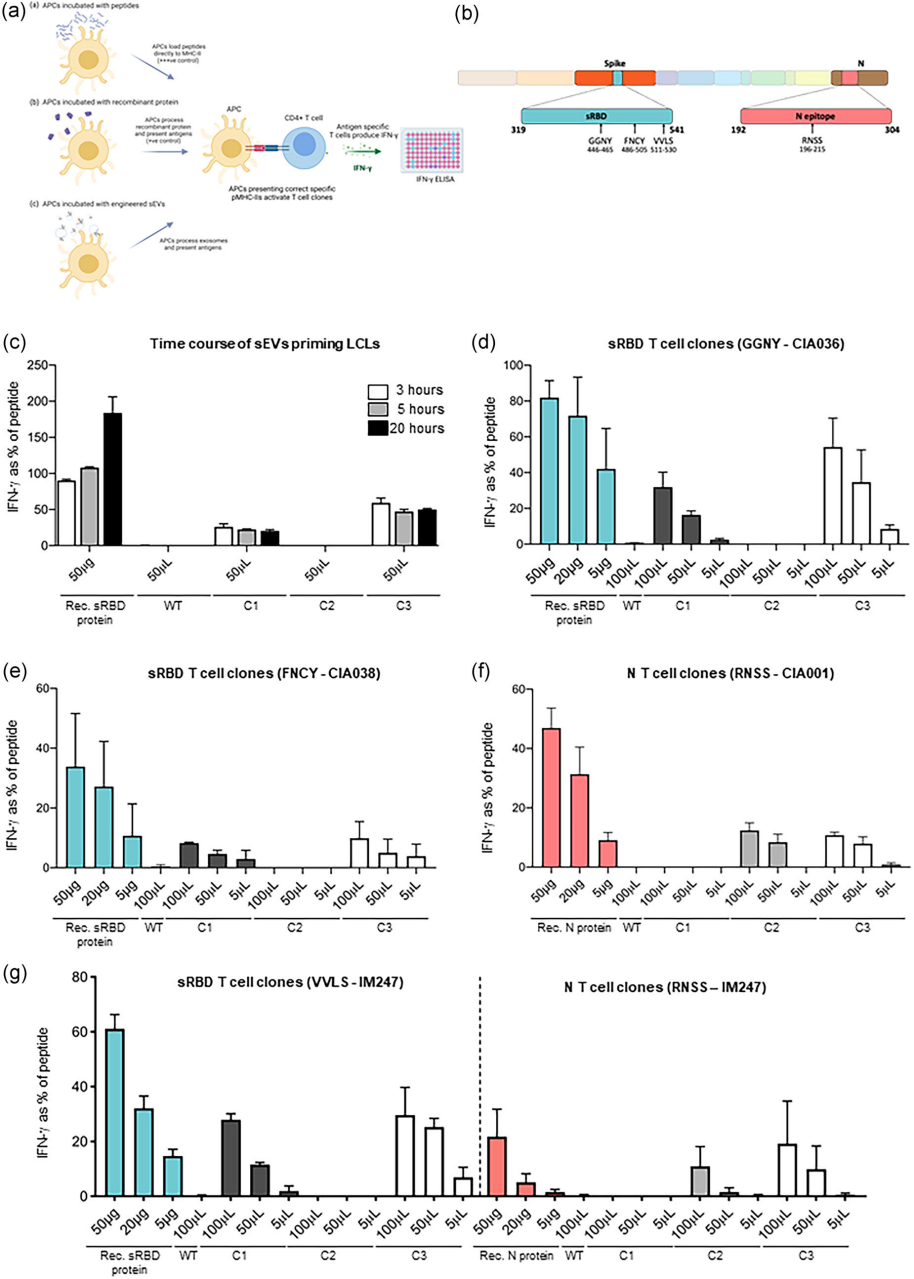
APCs primed with engineered sEVs can elicit recognition by T cell clones. (a) A schematic representation of the T cell assays utilised. B cell‐derived lymphoblastoid cell lines (LCLs) expressing relevant HLA class II restriction alleles were established as antigen presenting cells (APC). These were exposed to either pepmix (+++ ’ve control), recombinant sRBD or N protein (+’ve control) or purified sEVs, then washed and co‐cultured with T cell clones. After 18 h of co‐culture, the culture supernatant medium was harvested and the IFN‐γ release into the supernatant was tested by IFN‐γ ELISA. (b) Schematic representation of the domains in the SARS‐CoV‐2 genome organization. The regions included in our genetically engineered sEVs; sRBD (residues 319–541 of SARS‐CoV‐2 Spike) and an antigenic fragment of nucleocapsid (N) protein (residues 192–304), are highlighted. The epitopes recognized by the T‐cell clones specific for sRBD (GGNY, FNCY and VVLS) and N (RNSS) are also highlighted. (c) LCLs were incubated with peptide, recombinant sRBD protein or 100 μL sEVs for either 3, 5 or 20 h. LCLs were then washed before being co‐cultured with sRBD‐specific (epitope GGNY) T cell clones. IFN‐γ release was measured by ELISA and values normalised against the mean maximal response detected against the epitope peptide. *n* = 2; mean ± SEM. (D‐G) LCLs were treated as described for panel C, but for 3 h only, were washed and co‐cultured for 18 h with T cell clones specific for the following epitopes: (d) sRBD epitope GGNY, (e) sRBD epitope FNCY, (f) N epitope RNSS. (g) LCL IM247, which expresses the HLA class II restriction alleles for the sRBD epitope VVLS and the N epitope RNSS was incubated as described in panel C. After washing, the cells were co‐cultured for 18 h with either of the two T‐cell clones, quantifying the display of both epitopes from the same LCL. For panels C‐G, *n* = 3; mean ± SEM. Rec. protein: recombinant protein.

Human CD4+ T cell assays were established to assess the ability of bioengineered sEVs to deliver antigens to APCs for processing and presentation to T cells. Autologous B cell‐derived lymphoblastoid cell lines (LCL) were established as APCs, and LCLs were pulsed with either peptide, recombinant protein, mock sEVs, or bioengineered sEVs. After pulsing, LCLs were washed before being co‐cultured with CD4+ T‐cell clones overnight (Figure 3a). T cell activation was determined using IFN‐γ ELISA assays and the degree of T cell responses were normalized to maximal responses elicited against the control LCL loaded with epitope peptide. The CD4+ human T cell clones used for these experiments were isolated from SARS‐CoV‐2 convalescent healthcare workers and their epitope specificity was validated as previously described (Tye et al., 2022). T cell clones recognizing three SARS‐CoV‐2 sRBD epitopes (GGNY, FNCY and VVLS) and one Nucleocapsid epitopes (RNSS) were selected based on these epitopes being located within the recombinant CD63 constructs in the bioengineered sEVs (Figure 3b, Figure S3A).

To determine the optimal duration of antigen uptake, processing and presentation, LCLs were incubated with sEVs for 3, 5 or 20 h before being washed and co‐cultured with the sRBD T cell clone, GGNY, overnight (Figure 3c). LCLs incubated with C1 or C3 sEVs were capable of inducing IFN‐γ production from sRBD‐specific T cells, and there was no clear improvement of IFN‐γ response with longer incubation with C2 sEVs. We next evaluated the efficacy of T cell recognition of sEV‐delivered antigens by clones specific for different SARS‐CoV‐2 epitopes, using matched LCLs expressing the relevant HLA class II restriction alleles (Figure S3A). LCLs primed with C1 and C3 sEVs elicited a dose‐dependent stimulation of sRBD T cell clones GGNY and FNCY (Figure 3d, e). As expected, neither of the LCLs primed by mock or C2 sEVs that lack the sRBD fragment activated GGNY or FNCY T cells. Conversely, LCLs incubated with sEVs containing the Nucleocapsid region, C2 and C3, were able to activate the T cell clone RNSS (Figure 3f), but LCLs incubated with mock and C1 sEVs were not. T cell clones specific for epitopes within Spike or Nucleocapsid but *outside* the regions expressed on our sEVs were used as additional controls (Figure S3B). As expected, Spike SSAN T cells were activated by LCLs loaded with epitope peptide, but not by LCLs loaded with recombinant sRBD protein or sEVs (Figure S3C). T cells specific for the Nucleocapsid QTQH epitope were activated by LCLs incubated with peptide‐ and recombinant (full length) N protein but not LCLs incubated with sEVs (Figure S3D).

Of importance, we noted that C3 sEVs elicited dose‐dependent responses from T cells specific for either the sRBD (GGNY and FNCY) or N region (RNSS) when tested independently using separate LCL targets (Figure 3e, f). To determine whether multiple antigens can be simultaneously processed from sEVs for T cell recognition, we used an LCL (IM247) which expresses the HLAII restriction alleles DRB1*01:01 and DRB1*09:01 that present the sRBD VVLS and N RNSS epitopes, respectively (Figure S3A). As expected, IM247 LCL incubated with the single antigen‐expressing sEVs C1 and C2 evoked responses from sRBD VVLS and N RNSS T cell clones, respectively (Figure 3g). Importantly, IM247 LCL pulsed with the dual antigen‐expressing sEVs, C3, stimulated both sRBD VVLS and N RNSS T cell clones in the same assay, demonstrating the ability of sEVs to co‐deliver multiple antigens to APCs.

To test the stability of bioengineered sEVs, LCLs were pulsed with freshly isolated sEVs or with sEVs that had undergone 10 freeze‐thaw cycles. No significant differences were found in the degree of response when using either fresh or frozen sEVs (Figure S3E), demonstrating that sEV preparations retain antigenicity following freeze‐thaw cycles. The morphology of sEVs did not appear to be impacted by freeze‐thaw cycles and sEVs maintained their characteristic appearance by TEM (Figure S3F).

Collectively, these data demonstrate that sEVs can be internalized by APCs, and their antigens processed and presented via MHC‐II to CD4+ T cells. Additionally, these data confirm that sEVs can be used to co‐deliver antigens to drive multiple distinct immune responses.

### 3.5 Immunisation of mice with engineered sEVs stimulate B and T cells in vivo

**FIGURE 4:**
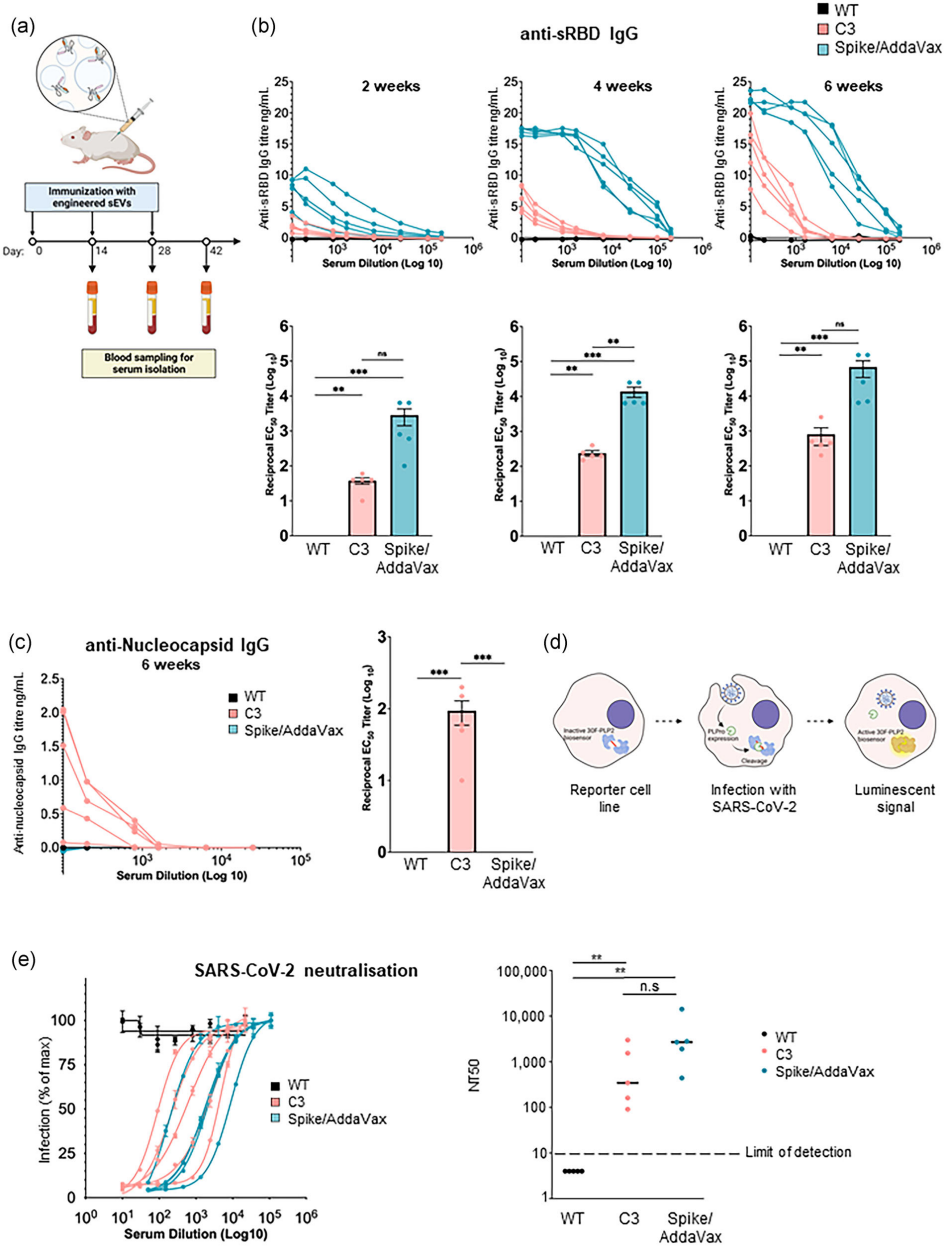
Vaccination with engineered sEVs stimulate B and T cells in vivo. (a) Schematic overview of the in vivo study design. C57BL/6 mice were immunised subcutaneously with either WT sEVs, C3 sEVs, or recombinant Spike protein adjuvanted with AddaVax (Spike/Addavax) three times with 2‐week intervals. Blood was collected 14, 28 and 42 days after the initial vaccination and serum was isolated for analysis. *n =* 5 mice per group. (b) Results of anti‐sRBD IgG ELISAs measuring sRBD‐specific IgG titres in serum samples taken 14, 28 and 42 days after vaccination. EC50s were calculated for each sample. *n =* 5. Significance was assessed by ordinary one‐way ANOVA analysis with Tukey's multiple comparison tests. (c) Results of an anti‐N IgG ELISA used to measure N‐specific IgG titres for serum samples after 6‐weeks. Data analysis was performed as described in panel B. (d) Schematic showing activation of luminescent HEK293T‐ACE2‐30F‐PLP2 reporter cells by SARS‐CoV‐2 infection. The inactive firefly luciferase (FFluc)‐based 30F‐PLP2 biosensor is cleaved by viral Papain‐like protease (PLPro), and infection quantified as FFluc luminescence. (e) Titration curves (left panel) and neutralising antibody titres at 50% inhibition (NT_50_s, right panel) for serum samples after 6‐weeks. *n =* 5. Horizontal bars, median values. Dotted line, limit of detection. Samples with no detectable neutralising activity are plotted at an arbitrary NT_50_ of 4. Significance was assessed using a Mann‐Whitney U‐test (non‐parametric data). ns = non‐significant, *p < 0.05, **p < 0.01, ****p < 0.001, ****p < 0.0001.

Having demonstrated that engineered sEVs can successfully stimulate T cells in vitro, we next aimed to determine whether they can effectively elicit in vivo immune responses. C57BL/6 mice were immunised subcutaneously with C3 sEVs on days 0, 14 and 28 (Figure 4a). Recombinant Spike protein adjuvanted with AddaVax (oil‐in‐water squalene solution) was employed as a positive control and WT sEVs were used as negative controls. sEVs were administered in PBS and without adjuvant. Blood was collected from mice on days 14, 28 and 42 and serum isolated for ELISA.

Two weeks after immunisation, sRBD IgGs were detectable from mice immunised with C3 sEV or Spike/AddaVax‐immunised mice, but not from mice immunised with WT sEVs (Figure 4b). sRBD IgG titres were lower from C3 sEV‐immunised mice versus Spike/AddaVax‐immunised mice. Serum collected 4 weeks after the initial immunisation (2 weeks after boost immunisation) had increased sRBD IgG from C3 and Spike/AddaVax immunised mice. sRBD IgGs were greatly increased again in the serum collected 6 weeks after initial immunisation. The serum from mice immunised with WT sEVs showed no detectable titre at any time point.

Since C3 sEVs contained both sRBD and a Nucleocapsid antigen, we also measured anti‐Nucleocapsid IgG titres. Only the serum from C3 immunised mice contained anti‐Nucleocapsid IgGs (Figure 4c), demonstrating the ability of C3 sEVs to generate both anti‐sRBD and anti‐Nucleocapsid IgGs. Importantly, mice did not show any adverse reactions to human‐derived sEVs.

T cell immunity plays a central role in infectious disease control. We therefore assessed the ability of sEVs to induce antigen‐specific T cell responses. For this purpose, cells of whole spleens obtained from the vaccinated C57BL/6 mice were stimulated *ex vivo* with sRBD peptide, sRBD pepmix or recombinant sRBD protein. Evaluation of sEV‐induced adaptive T cell responses was performed by assaying spleen cytokine IFN‐γ in the supernatants from spleen cell suspensions. T cell responses in mice immunised with C3 sEVs were consistently weaker compared to the positive control animals immunised with Spike/AddaVax, but larger than from mice immunised with mock sEVs where no IFN‐γ was detected (Figure S4A).

Neutralising antibodies generated by vaccination or natural infection bind to epitopes on the surface of viruses and impair their ability to engage host surface receptors, or impair conformational changes in viral proteins required for entry to host cells. We therefore assessed the ability of our bioengineered sEVs to produce antibodies capable of neutralising SARS‐CoV‐2 infection using a protease‐activatable luminescent reporter cell line (Gerber et al., 2022). These cells stably express an inactive luciferase‐based biosensor (30F‐PLP2), which is a substrate for SARS‐CoV‐2 Papain‐like protease (PLPro). Cleavage of the biosensor when cells are infected with virus liberates active firefly luciferase, which may be quantified by luminometry (Figure 4d). Clear sigmoidal neutralisation curves were observed in assays using serum from mice immunised with C3 sEVs or Spike/AddaVax (Figure 4e). Conversely, in agreement with anti‐sRBD IgG responses, we observed no detectable neutralising activity in mice immunised with WT sEVs. Of note, although anti‐sRBD IgG antibody titres obtained after sEVs immunisation were lower, the neutralising capacity was similar to those from Spike/AddaVax immunised mice. This highlights the high quality and efficacy of antibodies generated by sEVs vaccination.

Together, these data demonstrate that sEVs can be bioengineered to deliver dual viral antigens and that these sEVs are capable of generating IgG responses in vivo which neutralise virus infection.

## 4 DISCUSSION

EVs represent an exciting therapeutic modality to deliver drugs or proteins to cells within naturally derived vehicles and a number of studies have demonstrated sEVs as effective vaccine platforms (Sabanovic et al., 2021). sEVs can be genetically engineered to contain chimeric proteins, providing an opportunity to deliver multiple T cell epitopes within a single vesicle. To this end, we have genetically engineered single sEVs containing antigens from SARS‐CoV‐2 sRBD and an antigenic region of Nucleocapsid. sEVs containing sRBD or Nucleocapsid fragment—in isolation or in combination—were engineered to the inside or the outside of sEVs in accordance with their natural topology and had no impact on CD63 trafficking or localisation. These sEVs were able to prime APCs to activate antigen‐specific T cell clones and were able to stimulate the generation of IgGs in vivo, highlighting sEVs as efficient vaccines and platforms for vaccine development.

The humoral immune response to SARS‐CoV‐2 infection results in the generation of antibodies against Spike and Nucleocapsid (Li et al., 2020). Anti‐Spike antibodies neutralise SARS‐CoV‐2 and inhibit its replication, and all currently approved mRNA and AAV‐based COVID vaccines encode Spike or components of it. Anti‐Nucleocapsid antibodies are found in equal or higher amounts than anti‐Spike antibodies in SARS‐CoV‐2 convalescents (Moderbacher et al., 2020). Although raised against internal antigens and non‐neutralising, anti‐N antibodies are protective, and antibodies against internal antigens prevent infection by many viruses including Ebolavirus (Wilson et al., 2000), vaccinia virus (Moss, 2011) and HIV‐1 (Excler et al., 2014). How non‐neutralising antibodies such as anti‐Nucleocapsid antibodies provide immune protection remains largely unknown (Albecka et al., 2021). Although Spike‐based vaccines have proved extremely successful, the mutations within SARS‐CoV‐2 Spike have raised concerns for antibody escape mutants. One proposed solution is to generate vaccines encoding both Spike and Nucleocapsid, making it less likely for resistant viruses to emerge (Albecka et al., 2021; Burbelo et al., 2019; Dangi et al., 2021).

In order for bioengineered sEVs to prime LCLs or generate IgGs in vivo, APCs must present MHC:peptide complexes. APCs may acquire these MHC:peptide complexes by two potential routes; (1) bioengineered sEVs contain preformed MHC:peptide complexes which are delivered to cells, or (2) sEVs deliver antigens to APCs which are subsequently processed to peptides, loaded to MHC‐II, and presented by the APC.

In this study, sEVs were harvested from HeLa cells which do not express MHC‐II and as such are unable to deliver preformed MHC:peptide complexes. Given our bioengineered sEVs were able to evoke immune responses in vitro and in vivo, this demonstrates that sEVs were acquired by APCs, and that sEV‐derived antigens were processed, loaded to MHC‐II and displayed by APCs. Our study also demonstrates that sEV‐derived antigens are acquired, processed and presented by APCs irrespective of whether antigens are positioned on the inside or the outside of sEVs. This shows that sEVs are degraded within APCs to liberate antigens from inside of the sEVs.

Bioengineered sEVs were able to evoke similar levels of T cell stimulation with doses lower than that of recombinant protein. In human T cell assays, LCLs pulsed with 100 μL of either C1, C2 or C3 sEVs evoked similar levels of IFN‐γ release as LCLs pulsed with 5 μg recombinant protein (Figure 3c–h). Our data indicate that 100 μL of C1 sEVs contains between 148 and 299 ng of sRBD/FLAG, that 100 μL of C2 sEVs contain 890 ng FLAG, and that 100 μL of C3 sEVs contain 281–500 ng of sRBD/FLAG (and therefore also of Nucleocapsid antigen). These data show that sEVs are highly efficient vehicles for delivering antigens to APCs and are able to deliver antigen to stimulate immune responses at lower doses than by using recombinant protein. Extracellular vesicles are actively bound and internalised by cells (Prada & Meldolesi, 2016) and therefore are likely to be acquired preferentially by APCs. sEVs may also provide an environment to protect proteins from extracellular damage and degradation, improving the stability and half‐life of proteins.

Immunisation of mice with C3 sEVs induced the generation of anti‐sRBD and anti‐Nucleocapsid IgGs (Figure 4b, c). Although the anti‐sRBD antibody titres were lower than those of mice immunised with Spike/AddaVax, it is important to note that the quantity of Spike protein in the Spike/Addavax preparation (5 μg Spike) is much higher than the quantity of sRBD administered in the sEVs. Furthermore, the sEVs were administered without an adjuvant. We suspect that the addition of a suitable adjuvant to our sEVs may be able to drive a stronger immune response.

The ability to generate sEVs with multiple foreign antigens has other potential applications. Addition of protein domains that facilitate capture and uptake, or the ability to target specific cell types could be added. One limitation of CD63 as an sEV scaffold is that both N‐ and C‐termini are localised on the inside of the sEV and so cannot be engineered to contain exposed ligands or receptors to facilitate targeting. However, other sEV scaffolds may be explored to aid such targeting, such as PTGFRN or other single‐pass transmembrane proteins that are enriched in extracellular vesicles (Dooley et al., 2021).

One challenge in vaccine development is the production of appropriate and cost‐effective methods to test pre‐determined antigen candidates. Our work highlights sEVs to be appropriate and adaptable platforms for the co‐administration of vaccine candidates during in vitro or in vivo trials. Furthermore, their ease of engineering and tailoring, potentially allows sEV‐based vaccines to be used in personalised immunotherapy, for example, for certain cancers.

In summary, our findings demonstrate that sEVs hold significant promise as versatile platforms for delivering multiple antigens, generating immune responses, and potentially advancing personalized immunotherapy. While further advancements are needed to overcome production challenges, sEVs offer adaptable and cost‐effective methods for pre‐clinical testing of vaccine candidates, making them valuable tools in vaccine development.

## AUTHOR CONTRIBUTIONS

**Hannah K Jackson**: Data curation; investigation; formal analysis; methodology; software; writing—original draft; writing—review and editing. **Heather M. Long**: Investigation; formal analysis; methodology; resources; writing—review and editing. **Juan Carlos Yam‐Puc**: Investigation; formal analysis; writing—review and editing. **Roberta Palmulli**: Investigation; writing—review and editing. **Tracey A. Haigh**: Investigation; project administration. **Pehuén Pereyra Gerber**: Investigation; formal analysis; methodology; writing—review and editing. **Jin S. Lee**: Investigation; formal analysis. **Nicholas J. Matheson**: Project administration; writing—review and editing. **Lesley Young**: Project administration; writing—review and editing. **John Trowsdale**: Project administration; writing—review and editing. **Mathew Lo**: Project administration. **Graham S. Taylor**: Project administration; writing—review and editing. **James E. Thaventhiran**: Project administration; writing—review and editing. **James R. Edgar**: Conceptualization; data curation; investigation; formal analysis; funding acquisition; methodology; project administration; resources; software; supervision; writing—original draft; writing—review and editing; visualization; validation.

## CONFLICT OF INTEREST STATEMENT

The authors declare no conflicts of interest.

## Supporting information

Supplementary Information

Supplementary Information

Supplementary Information

Supplementary Information

## Data Availability

All data are available in the manuscript or the supplementary materials.
