# Supplementary figures and images for: Bioengineered small extracellular vesicles deliver multiple SARS‐CoV‐2 antigenic fragments and drive a broad immunological response
Source: J Extracell Vesicles. 2024 Feb 9;13(2):e12412. doi: 10.1002/jev2.12412 (PMC10858312; doi:10.1002/jev2.12412)

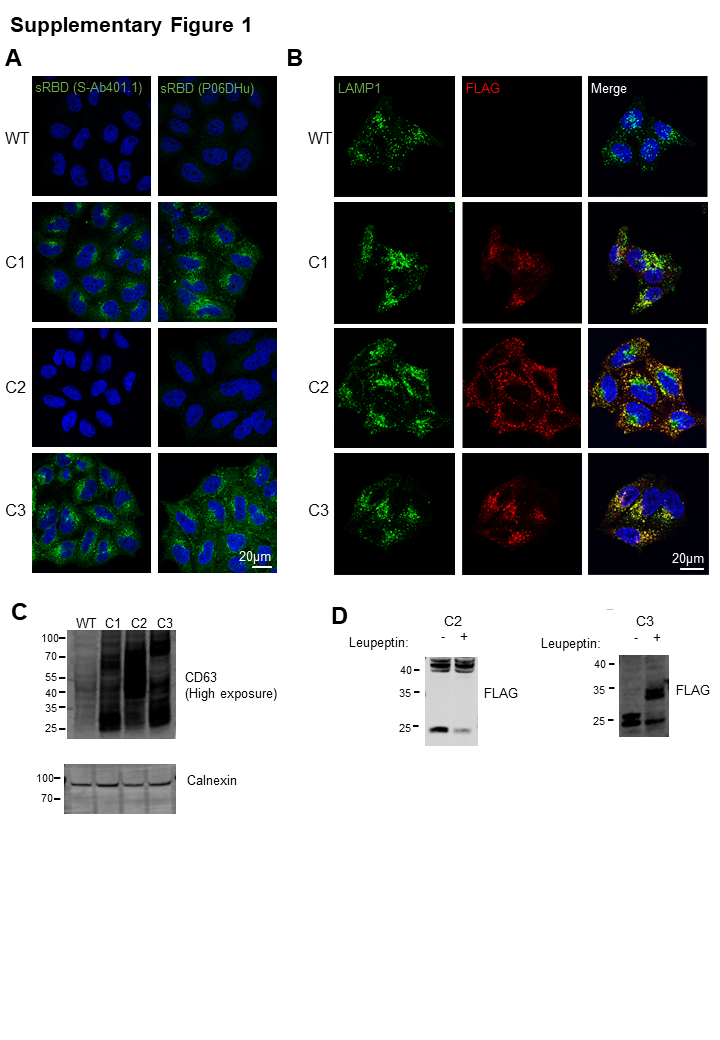

Supplement: Supplementary file 1 — Supplementary Information [file JEV2-13-e12412-s004.TIF]

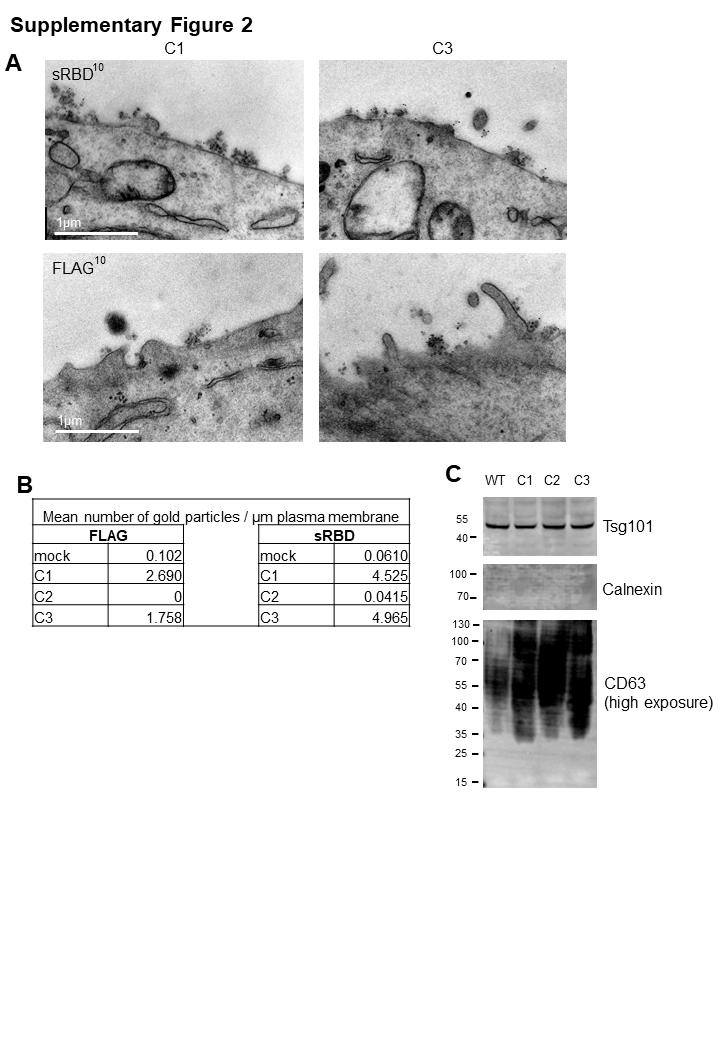

Supplement: Supplementary file 2 — Supplementary Information [file JEV2-13-e12412-s002.TIF]

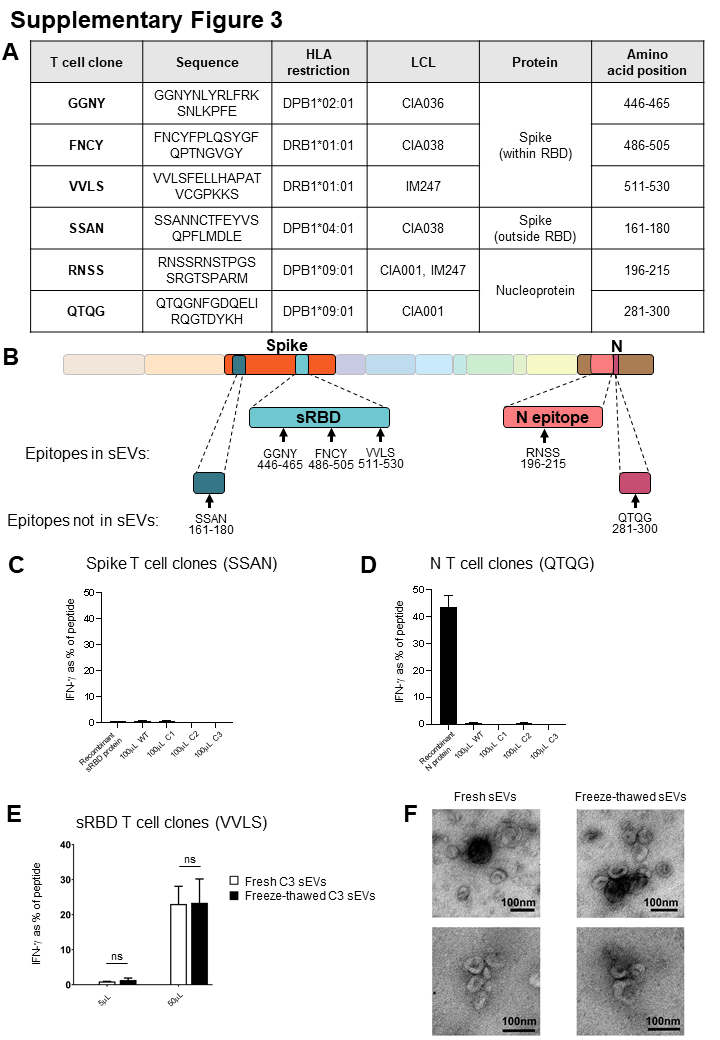

Supplement: Supplementary file 3 — Supplementary Information [file JEV2-13-e12412-s003.TIF]

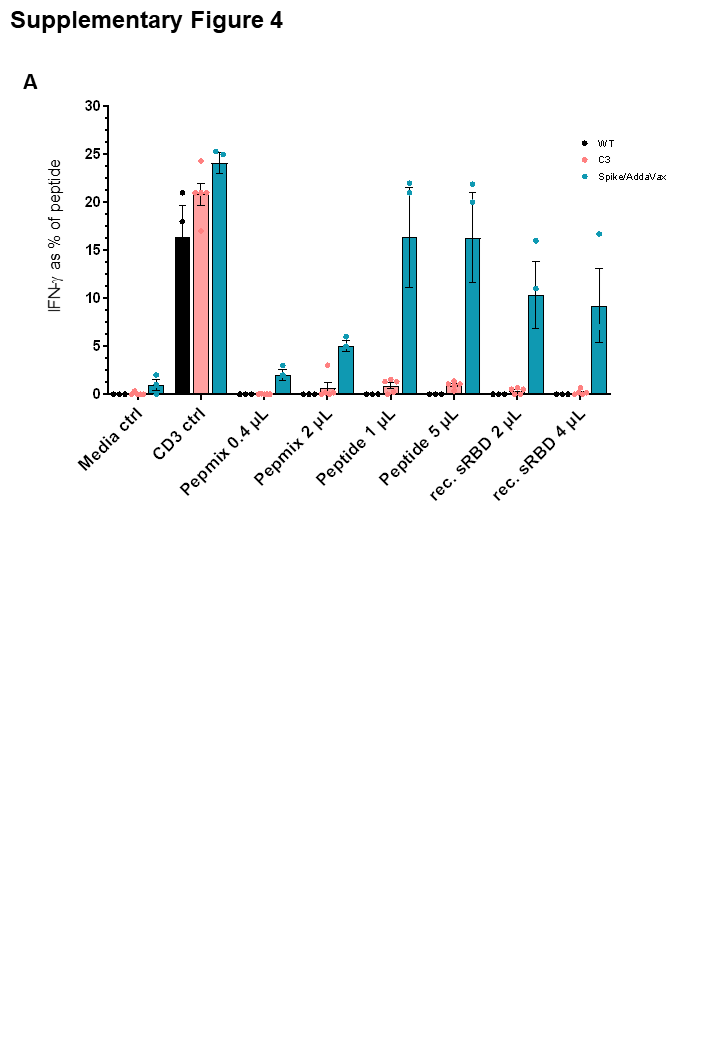

Supplement: Supplementary file 4 — Supplementary Information [file JEV2-13-e12412-s001.TIF]
